# Althæa Officinalis

**Published:** 1884-06

**Authors:** F. C. Berry


### ALTHÆA OFFICINALIS.

The *Althœa officinalis,* or marsh mallow, belongs to the natural order *Malvaceœ,* and is to be found growing plentifully in most European countries. On the Continent, especially in France, it is highly esteemed for its demulcent properties. Both the leaves and roots are used, and are given internally for various affections of the mucous membranes. A favorite remedy in France for sore throat is *pate de guimauve,* which is a kind of lozenge made with mucilage of althæa, gum-arabic, sugar, and white of egg. Recipes for a decoction and sirup of althæa were to be found in the old London pharmacopœia. In this part of the country the ointment is the favorite preparation, and is made by cutting the fresh leaves into small pieces, stirring them together with lard, and boiling the mixture for half an hour, after which process it is strained through muslin or through a common kitchen strainer, and is then ready for use.—F. C. BERRY in *Birm. Med. Review.*

This drug has also been used in treatment of eczema. A proving might bring forth new virtues.—EDITOR.

